# Total flavonoids from *Abelmoschus manihot* (L.) improve diabetes nephropathy by regulating the gut–kidney axis

**DOI:** 10.3389/fmed.2025.1693643

**Published:** 2026-01-28

**Authors:** Hongmei Yu, Yuanxin Liu, Harvest F. Gu, Wei Tang, Nan Li

**Affiliations:** 1 College of Pharmacy, Qilu Medical University, Zibo, China; 2 Islet Cell Senescence and Function Research Laboratory, Department of Endocrinology, Nanjing Medical University Affiliated Geriatric Hospital/Jiangsu Province Geriatric Hospital, Nanjing, China; 3 Laboratory of Molecular Medicine, School of Basic Medicine and Clinical Pharmacy, China Pharmaceutical University, Nanjing, China; 4 Department of Endocrinology, Jiangsu Province Hospital of Chinese Medicine, The Affiliated Hospital of Nanjing University of Chinese Medicine, Nanjing, China

**Keywords:** diabetic nephropathy, gut–kidney axis, irbesartan, total flavones of *A. manihot* (L.) Medic, type 2 diabetes

## Abstract

**Background:**

A recent clinical study demonstrated that Huangkui capsule (with its bioactive constituents being total flavones extracted from *Abelmoschus manihot* (L.), TFA) combined with irbesartan provides effective therapy for type 2 diabetes (T2D) patients with diabetic nephropathy (DN).

**Objective:**

This study aimed to elucidate the therapeutic mechanisms of TFA in DN through the modulation of the gut–kidney axis.

**Methods:**

The db/db mice were administered TFA, irbesartan, or vehicle. Urinary albumin-creatinine ratio (UACR) was measured by the enzyme-linked immunosorbent assay (ELISA). Intestinal bacterial composition was analyzed using 16S rRNA sequencing. Serum metabolites were quantified via LC-ESI-MS/MS. Kidney transcriptomics were assessed using Illumina platform-based RNA sequencing.

**Results:**

Administration of TFA reduced the UACR in db/db mice and significantly altered intestinal flora composition. Specifically, TFA elevated the abundance of Dietzia, Faecium, Streptococcus, and Blautia while reducing Bacteroidetes, Firmicutes, Enterobacteriaceae, Rikenellaceae, Fusivibrio, and Treponema. In serum metabolomic analysis, TFA increased the levels of quercetin 3-glucuronide and n-cinnamyl glycine but decreased cortisol concentrations. Concurrently, renal transcriptomics revealed the downregulation of key genes, including retnlg, ngp, mpo, camp, ctsg, elane, s100a8, s100a9, trem1, and mmp7, which primarily function in pathways related to neutrophil extracellular trap formation, steroid hormone biosynthesis, and cortisol synthesis/secretion. In contrast, irbesartan treatment did not significantly affect blood pressure or specific renal gene pathways in db/db mice.

**Conclusion:**

TFA attenuates diabetic nephropathy (DN) progression through pharmacological mechanisms involving three key axes: (1) modulation of intestinal flora composition, (2) regulation of circulating metabolites, and (3) suppression of renal gene activity pathways. These findings highlight the gut–kidney axis as a central therapeutic target for TFA in DN management.

## 1 Introduction

Diabetic nephropathy (DN) persists as a predominant microvascular complication of diabetes mellitus, accounting for substantial global disease burden and mortality. Epidemiological studies demonstrate a parallel escalation in DN incidence with the worldwide diabetes pandemic (1, 2), particularly evident in China’s rapidly growing case numbers (3, 4). As the primary etiology of end-stage renal disease (ESRD) necessitating renal replacement therapy (5), DN underscores the urgent need for optimized therapeutic interventions.

The gut microbiota–host crosstalk plays a critical role in various diseases, including chronic kidney disease (CKD) and hypertension, which are associated with immune dysregulation, metabolic disorders, and sympathetic activation linked to gut dysbiosis. Evidence has demonstrated that the brain–gut–kidney axis exists in CKD and hypertension, proposing its role in disease pathogenesis and potential as a therapeutic target. Understanding this axis could uncover novel treatments, leveraging microbial metabolites and signaling pathways to address these conditions (6). Similarly, type 2 diabetes mellitus (T2D), characterized by hyperglycemia and multi-organ dysfunction, is linked to gut microbiota alterations. Using a db/db mouse model, Torrez Lamberti et al. (7) have reported that the probiotic *Lactobacillus johnsonii* N6.2 (LJN6.2) administration improved glycemic control, reduced diabetes severity, and mitigated pancreatic, hepatic, and renal damage. Additionally, LJN6.2-derived extracellular vesicles reduced hepatic lipid accumulation. These findings highlight LJN6.2’s promise as an adjuvant therapy for T2D, supported by microbiome profiling via 16S rRNA sequencing. These two studies underscore the gut microbiota’s pivotal role in CKD and T2D-DKD, offering avenues for innovative therapies targeting the brain–gut–kidney axis and microbial interventions.

The diagnostic paradigm of DN centers on persistent albuminuria and progressive glomerular filtration rate deterioration, establishing albuminuria reduction as a critical treatment endpoint (8, 9). Huangkui capsule (HKC), a standardized botanical drug extracted from *Abelmoschus manihot* (L.) Medik., has exhibited significant renoprotective effects in DN management through albuminuria attenuation (10). Clinical evidence from a multicenter randomized controlled trial (RCT) substantiates that HKC-irbesartan (EB) combination therapy significantly reduces proteinuria in patients with DN. Pharmacological investigations identify total flavones of *A. manihot* (TFA)—comprising rutin, hyperoside, isoquercitrin, gossypetin-8-O-β-D-glucuronide, hibifolin, myricetin, quercetin 3-O-β-D-glucuronide, and quercetin (10–12)—as bioactive constituents that mediate renal tubular solute carrier (SLC) modulation (13, 14).

Extending our prior findings in db/db murine models, which delineated HKC + EB’s multidimensional effects on gut microbiome dynamics, systemic metabolism, and renal gene networks (15), the present investigation specifically deciphers TFA as well as EB within the gut–kidney axis. Through integrated multi-omics analysis encompassing fecal microbiota sequencing, serum metabolomics, and renal transcriptomic profiling, this study systematically elucidates the mechanistic pathways of TFA from *A. manihot* in DN management via gut–kidney crosstalk regulation.

## 2 Methods

### 2.1 Animals

Db/db (BKS.Cg-Dock7m +/+ Leprdb/J) and C57BL/KsJ mice (10 weeks old) were obtained from the Animal Experimental Center of Nanjing University (Nanjing, China). All male mice were housed in a specific pathogen-free (SPF) barrier facility at the Animal Experimental Center, China Pharmaceutical University. The animal room was maintained at 24 ± 2 °C with 50 ± 10% humidity under a 12-h light/dark cycle. After 1 week of acclimatization, body weight and blood glucose levels were measured weekly. Urine samples were collected for 6 h using metabolic cages (DXL-XS, Fengshi, Suzhou, China). Microalbuminuria and creatinine levels were quantified using ELISA kits (Elabscience Biotechnology, China). Db/db mice were considered diabetic nephropathy (DN) models when meeting 2 consecutive days of: (1) blood glucose ≥16.7 mmol/L and (2) urinary albumin-to-creatinine ratio (UACR) ≥ 200 ng/μg. DN mice were then randomly allocated into six groups: untreated DN group (*n* = 15); TFA-treated group (TFA, *n* = 12); EB-treated group (EB, *n* = 6); TFA + EB combination group (TFA-EB, *n* = 10); and wild-type control (WT, C57BL/KsJ mice, *n* = 20).

### 2.2 TFA preparation and administration

TFA was prepared from ethanol extracts of *A. manihot* (L.) and provided by Suzhong Pharmaceutical Group Co., Ltd. (Taizhou, China). Each gram of TFA contained 2.4 mg of rutin, 189.6 mg of hyperoside, 188.7 mg of hibifolin, 142.9 mg of isoquercetin, 33.2 mg of myricetin, 29.4 mg of quercetin, 133.0 mg of quercetin-3-O-robinobioside, and 2.7% water. EB was manufactured by Sanofi Shengdelabao Minsheng Pharmaceutical Co., Ltd. (Hangzhou, China). Based on human–mouse body surface area conversion, db/db mice received a daily oral gavage of either TFA (75.50 mg/kg/d), EB (0.0195 g/kg/d), or vehicle control for 4 weeks, following our previously published protocol (12).

### 2.3 Renal histopathological analysis

By using the standard hematoxylin and eosin (H&E) staining protocol, histopathological analyses were conducted on kidney sections from mice in the control (Cont), diabetic nephropathy (DN), and TFA treatment groups. Following the collection of whole kidney tissues, they were meticulously embedded in paraffin using a routine embedding procedure. Subsequently, the paraffin-embedded blocks were sectioned into 4-μm-thick slices using the HistoCore Bio-Cutter (Leica Biosystem, Germany). The kidney sections were then subjected to staining with H&E reagents (BASO, Zhuhai, China) and meticulously examined under a CX23 light microscope (Olympus, Japan). For each individual mouse, five or more sections of the kidney were carefully selected for semi-quantitative analysis. Utilizing the Image-Pro Plus software (version 6.0.0260), precise measurements were taken to determine the glomerular area, vacuolar area, and kidney tubule diameter.

### 2.4 16S ribosomal DNA sequence analysis of gut microbiota

In the present study, the colons from mice were taken by surgical double ligation as described previously (16). Total gDNA from the colon microbiota was extracted using the CTAB/SDS method and then amplified by polymerase chain reaction (PCR) using primers 341F and 806R belonging to the V3–V4 variable regions of 16S rDNA. The mixed PCR products were detected with 2% agarose gel electrophoresis. The samples with main band brightness between 400 and 450 bp were selected for further experiments. The amplicons were purified using the AxyPrep DNA Gel Extraction Kit (Axygen Biosciences, Union City, CA, United States). After the DNA libraries were built using the NEB Ultra DNA Library Prep Kit (NEB), the 16S RNA sequences were analyzed using the NovaSeq 6000 (Illumina, San Diego, CA, United States) and Silva databases.

### 2.5 Metabolomic analysis of serum

The serum samples of mice were added to 20% of 300 μL acetonitrile carbinol (ACN: methanol = 1:4, V/V) and vortexed for 3 min. The mixture was then centrifuged for 10 min at 12000 r/min at 4 °C. The collection was centrifuged at 12,000 r/min for 3 min after being left at 20 °C for 30 min. The qualitative analysis of metabolites in serum was initially done with untargeted metabolomics in the Liquid Chromatography-Quadrupole Time-of-Flight Tandem Mass spectrometry platform and further adopted to the widely targeted metabolomics with the Liquid Chromatography-Electrospray Ionization Tandem Mass spectrometry system.

### 2.6 Transcriptomic analysis of kidney tissues

Total RNAs from kidney tissues were extracted using a Trizol reagent kit (Ambion, Shanghai Yubo Biological Technology Co., Ltd., China). The concentration of total RNAs was detected using the Qubit^®^ RNA Assay Kit (Life Technologies, CA, United States). The integrity of total RNAs was analyzed using the Nano 6000 of the Bioanalyzer 2100 System (Agilent Technologies, CA, United States). Library construction of total RNAs >1 μg was performed using the NEBNext^®^ Ultra^™^ RNA Library Prep Kit (Illumina, NEB, United States). Oligo(dT) beads were enriched with mRNA with a polyA tail, and mRNA was randomly interrupted in NEB fragmentation buffer. The cDNA was synthesized in the M-MuLV reverse transcriptase system (Illumina, NEB, United States) using the fragmented mRNA as a template and random oligonucleotides as primers. The cDNAs of approximately 200 bp were screened using AMPure XP beads (Beckman Coulter, Beverly, United States). The library was quantitated using a Qubit2.0 Fluorometer, and the insert size was detected using an Agilent 5400 Bioanalyzer (Agilent, United States). Once qualified, the library was sequenced on the Illumina platform (Illumina, Novo Geno Bioinformatics Co., Ltd., China).

### 2.7 Statistical analysis

All analyses and graphics were performed using GraphPad Prism software (version 8.0), SPSS software (version 23.0), and R software (version 3.6.0). Principal coordinate analysis (PCoA) was used for Adonis’s multivariate analysis of variance. Linear discriminant analyses of the effect size (LEfSe) between the two groups were tested for significance using the Kruskal–Wallis sum-rank test. Biological significance was subsequently analyzed using a set of pairwise tests among the groups using the Wilcoxon rank-sum test. Furthermore, the genus levels of gut microbiota were applied in the MetaStats statistical analysis. The integrating pathway enrichment and pathway topology analyses were conducted using MetaboAnalyst. The correlations between gut microbiota, metabolites, and differentially expressed genes were analyzed using Spearman’s correlation test. The comparisons between the groups were calculated using Student’s *t*-test. All values are presented as the means ± SEM unless otherwise noted. A *p*-value of <0.05 was considered statistically significant.

## 3 Results

### 3.1 Reduction of the UACR after treating with TFA, EB, or combination

**Figure 1:**
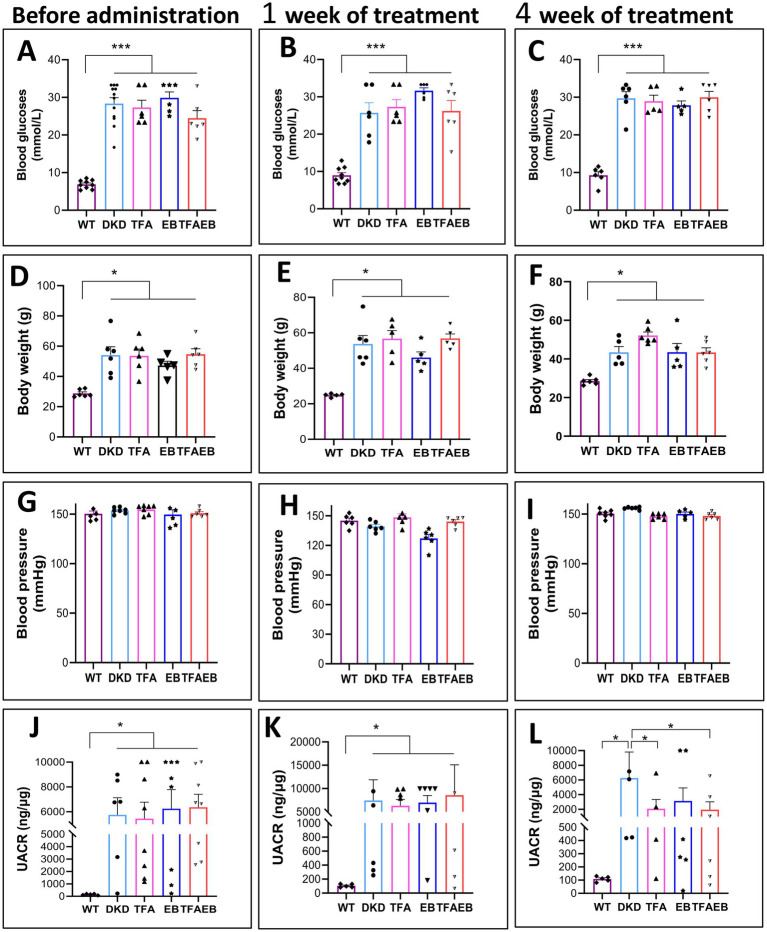
Changes in BG, BW, BP, and UACR after the treatment of TFA, EB, and their combination. Before TFA, EB, and combined drug administration and after administration for 1 week and 4 weeks, there was no significant change in BG, BW, and BP **(A–I)**. After 1 week of treatment, there was no significant reduction in the UACR **(J,K)**. After 4 weeks of treatment, however, the UACR was significantly reduced **(L)**. DN, diabetic nephropathy; WT, non-diabetic control; TFA, total flavones of *Abelmoschus manihot* (L.); EB, irbesartan; TFAEB, TFA combined with EB; UACR, urinary albumin-to-creatinine ratio; ^*^p < 0.05, ^**^p < 0.01, and ^***^p < 0.001.

Consistent with our previous reports (11, 12), the current study showed significantly elevated blood glucose (BG), body weight (BW), blood pressure (BP), and UACR in DN mice compared to the WT control group. Notably, TFA, EB, and their combination therapies significantly reduced the UACR in DN mice (Figures 1J–L), despite showing no statistically significant different changes in BG, BW, or BP after treating with TFA (Figures 1A–I).

### 3.2 Pathological changes (H&E staining) in renal tissues following administration‌

**Figure 2:**
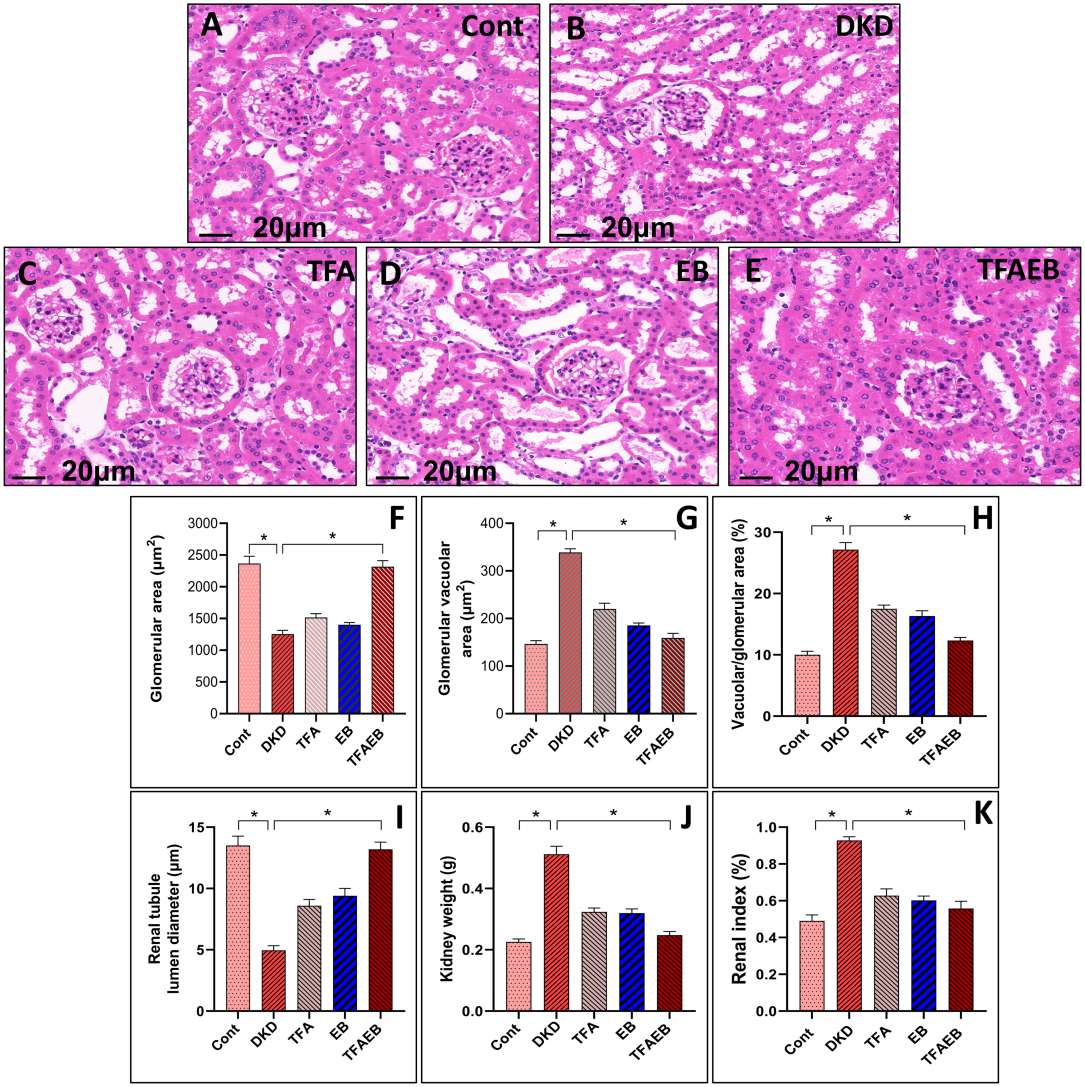
Pathological changes (H&E) of kidney tissues after drug administration. Representative photomicrographs **(A–E)** illustrate the pathological changes in renal tissues after the administration of TFA, EB, or their combination (TFAEB group), including the WT, DN, TFA, EB, and TFA-EB groups. Semi-quantitative comparative analysis **(F–I)** evaluates glomerular area, lumen diameter of renal tubules, and glomerular vacuole area across these groups, with data presented as mean ± SEM (*n* = 6 per group). Additionally, kidney weight and kidney index (kidney weight/body weight × 100%) are provided for all experimental groups **(J,K)**. DN, diabetic nephropathy; WT, non-diabetic control; TFA, total flavones of *Abelmoschus manihot* (L.); EB, irbesartan; TFAEB, TFA combined with EB.

Representative photomicrographs (A–E) illustrate the pathological changes in renal tissues following the administration of TFA, EB, or their combination, including the WT, DKD, TFA, EB, and TFA-EB groups. Semi-quantitative comparative analysis (F–I) evaluated key renal parameters—glomerular area, renal tubular lumen diameter, and glomerular vacuole area—across all experimental groups, with data presented as mean ± SEM (*n* = 6 per group). Key pathological and functional findings included the DKD group exhibiting significant renal hypertrophy, evidenced by increased kidney weight and kidney index (kidney weight/body weight × 100%) (J–K), consistent with DKD progression. Treatment with TFA, EB, or their combination mitigated DKD-induced renal damage. Notably, the TFA-EB group exhibited the most pronounced reduction in kidney weight and kidney index, suggesting a synergistic therapeutic effect. Compared to DKD, drug-treated groups showed reduced glomerular hypertrophy, preserved tubular architecture, and decreased glomerular vacuolation, as quantified in panels F-I. All quantitative data were analyzed using one-way ANOVA with *post-hoc* Tukey’s test. These findings highlight the renoprotective effects of TFA and EB, with their combination (TFA-EB) offering superior benefits in ameliorating DKD-associated structural and functional renal abnormalities (see Figure 2).

### 3.3 Gut microbiota composition after TFA, EB, or combined therapy

**Figure 3:**
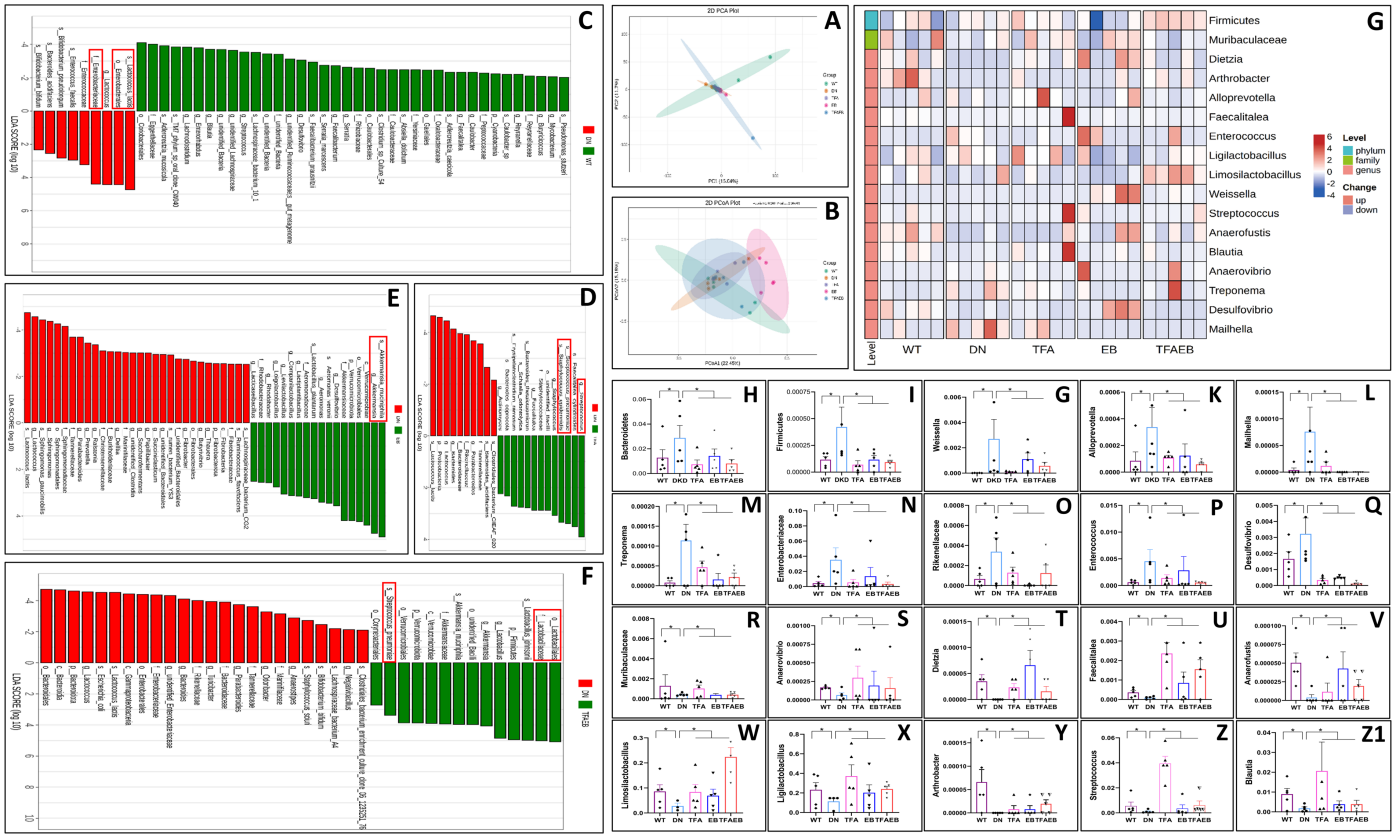
Effects on the intestinal flora after TFA, EB, and their combined administration. PCA and PCoA analysis of gut microbiota **(A,B)**; LEfSe analysis revealed the significant enrichment of WT, TFA, EB, and TFAEB compared with DN **(C–F)**; the clustering heatmap of OTU analysis demonstrated the change of gut microbiota in the DN, TFA, EB, and TFAEB groups **(G)**; quantify the OTU abundance of changes in the bacterial community after administration of each group based on the results of the clustering heatmap **(H–Z1)**. DN, diabetic nephropathy; WT, non-diabetic control; TFA, total flavones of *Abelmoschus manihot* (L.); EB, irbesartan; TFAEB, TFA combined with EB; ^*^p < 0.05.

Comparative analysis of gut microbiota among WT, DN, and treatment groups (TFA, EB, and TFA-EB) revealed distinct microbial profiles through principal component analysis (PCA) and PCoA (Figures 3A,B). PCA and PCoA revealed that the dispersion of the intestinal flora in the TFA and TFAEB groups is low. Compared with the TFA and TFAEB groups, the EB group showed a significant difference in the degree of gut microbiota dispersion. LEfSe analysis revealed significant enrichment of *Lactococcus lactis*, Enterobacterales, and Enterobacteriaceae in the DN group compared to the WT control group (Figure 3C). TFA treatment specifically increased the abundance of Streptococcus, Streptococcus, pneumoniae, and *Staphylococcus epidermidis* (Figure 3D). The EB group showed marked elevation of Akkermansia (Figure 3E), while combined TFA + EB treatment significantly enhanced Lactobacillales, Lactobacillaceae, and *Streptococcus pneumoniae* populations (Figure 3F). Based on Operational Taxonomic Unit-level (OTU) analysis, the clustering heatmap (Figure 3G) demonstrated that compared to the WT control group, the DN group exhibited increased abundance of Bacteroidetes, Firmicutes, Weissella, Alloprevotella, Mailhella, Treponema, Enterobacteriaceae, Rikenellaceae, Enterococcus, and Desulfovibrio—all of which were subsequently reduced by TFA, EB, or combination therapy (Figures 3H–Q). Conversely, Muribaculaceae, Anaerovibrio, Dietzia, Faecalitalea, Anaerofustis, Ligilactobacillus, Limosilactobacillus, Arthrobacter, Streptococcus, and Blautia showed decreased abundance in DN mice, with all treatment modalities effectively restoring these microbial populations (Figures 3R–Z1).

### 3.4 Serum metabolic profile changes induced by TFA, EB, or both

**Figure 4:**
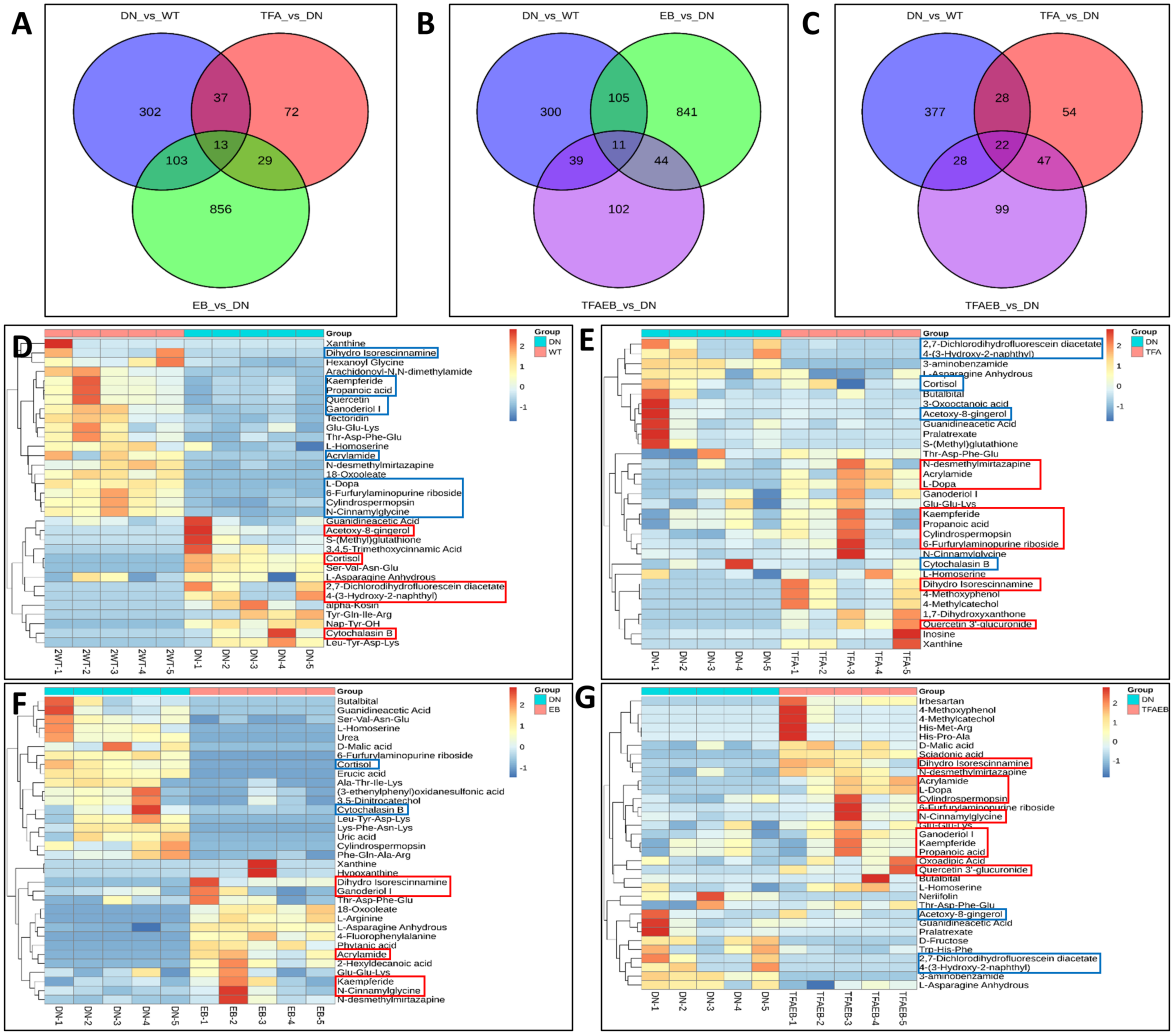
Alteration of serum metabolites after the treatment of TFA, EB, and their combination. Venn diagrams revealed common and distinct metabolites in serum among the WT, DN, TFA, EB, and TFAEB groups **(A–C)**. Cluster heatmaps demonstrated differential metabolite profiles between the DN, TFA, EB, and TFAEB groups **(D–G)**. DN, diabetic nephropathy; WT, non-diabetic control; TFA, total flavones of *Abelmoschus manihot* (L.); EB, irbesartan; TFAEB, TFA combined with EB. The red box indicates upregulation, while the blue box means downregulation.

The Venn diagrams display the common and distinct metabolites in serum among the DN, TFA, and EB groups (Figure 4A); DN, EB, and TFAEB groups (Figure 4B); and DN, TFA, and TFAEB groups (Figure 4C). Cluster heatmaps demonstrated differential metabolite profiles between the DN and other experimental groups (Figures 4D–G). Comparative analysis showed significant upregulation (red boxes) of cortisol, acetoxy-8-gingerol-4-(3-hydroxy-2-naphthyl)-2-oxobut-3-enoic acid, and 2,7-dichlorodihydrofluorescein diacetate in the DN group compared to the WT control group. Conversely, L-dopa, acrylamide, dihydro isorescinnamine, N-cinnamylglycine, quercetin, ganoderiol I, propanoic acid, and kaempferide were downregulated (blue boxes) (Figure 4D). TFA treatment induced significant increases in kaempferide, ganoderiol I, N-CinnaMylglycine, dihydro isorescinnamine, acrylamide, quercetin 3′-glucuronide, and L-dopa while decreasing cortisol, 4-(3-hydroxy-2-naphthyl)-2-oxobut-3-enoic acid, 2,7-dichlorodihydrofluorescein diacetate, and acetoxy-8-gingerol (Figure 4E). The EB group specifically upregulated kaempferide, ganoderiol I, dihydro isorescinnamine, and acrylamide while downregulating cortisol (Figure 4F). Combined TFA + EB treatment exhibited synergistic effects, elevating kaempferide, ganoderiol I, quercetin 3′-glucuronide, N-CinnaMylglycine, dihydro isorescinnamine, and acrylamide while reducing 4-(3-hydroxy-2-naphthyl)-2-oxobut-3-enoic acid, 2,7-dichlorodihydrofluorescein diacetate, and acetoxy-8-gingerol (Figure 4G). These findings collectively suggested that both individual and combined treatments induce specific metabolic reprogramming in the experimental model.

### 3.5 KEGG pathway analysis of serum metabolomic alterations

**Figure 5:**
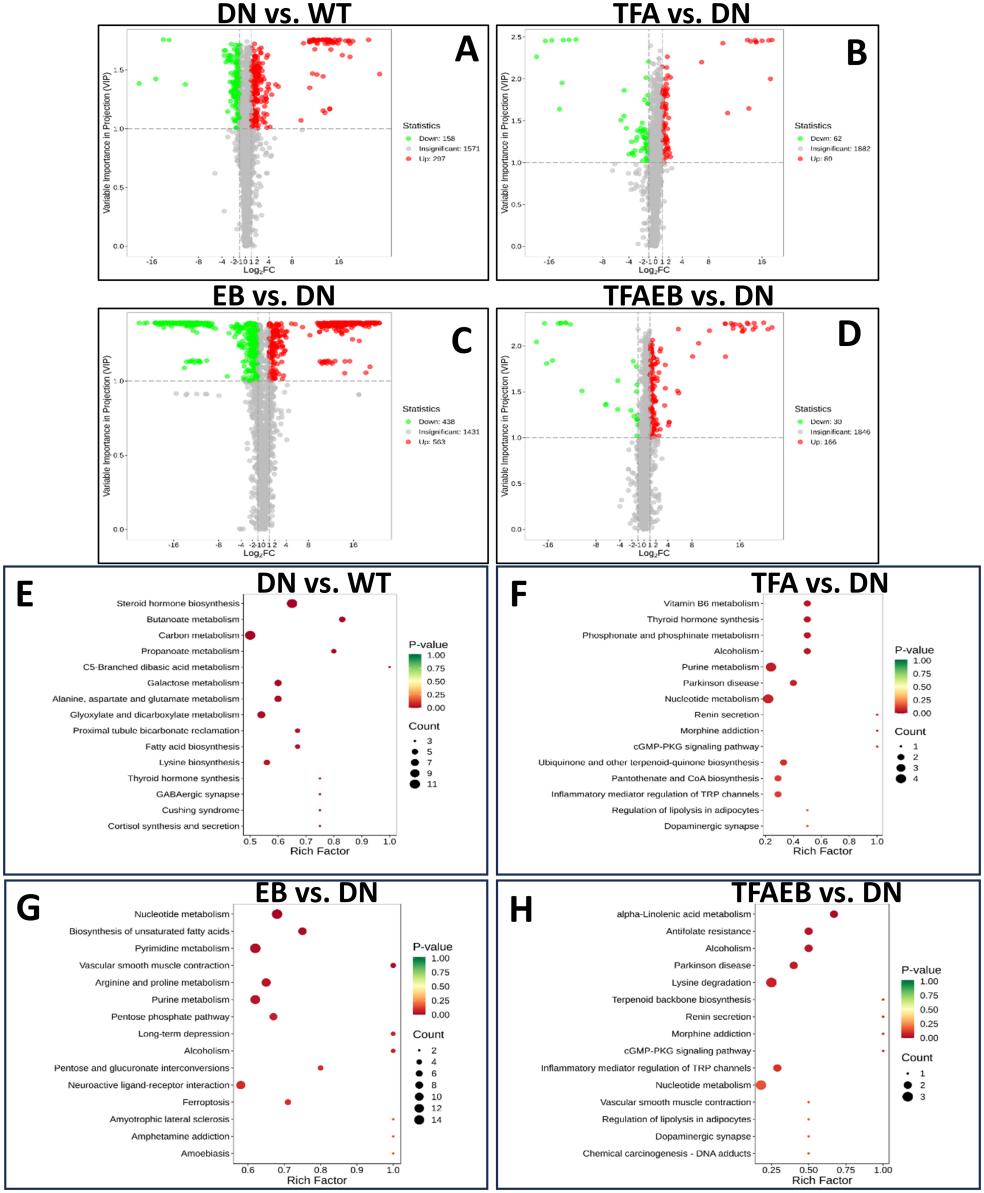
Volcano plot displays a KEGG pathway enrichment analysis of differential metabolites within each group. Volcano plots of serum differential metabolites between DN vs. WT, TFA vs. DN, EB vs. DN, and TFAEB vs. DN groups **(A–D)**. KEGG pathway enrichment analyses of the differentially expressed metabolites in kidneys **(E–H)**. According to VIP ≥1.0, *p* ≤ 0.05 screening differential metabolism. DN, diabetic nephropathy; WT, non-diabetic control; TFA, total flavones of *Abelmoschus manihot* (L.); EB, irbesartan; TFAEB, TFA combined with EB.

The volcano plot displays the common and distinct metabolites in serum among the groups. Compared with the WT group, the DN group had 297 metabolites that were upregulated and 158 metabolites were downregulated (Figure 5A). Compared with the DN group, the TFA group had 89 metabolites that were significantly elevated and 62 metabolites were significantly downregulated (Figure 5B). After the EB treatment, 563 metabolites were upregulated, and 438 metabolites were downregulated (Figure 5C). With the TFA and EB combination treatment, the 166 metabolites significantly increased, and the 30 metabolites significantly down-regulated (Figure 5D). KEGG pathway enrichment analysis of differential metabolites was performed using the ClusterProfiler package in R. Significantly enriched pathways in the DN group included steroid hormone biosynthesis, carbon metabolism, and butanoate metabolism (Figure 5E). The TFA treatment group showed remarkable enrichment in purine metabolism, Parkinson’s disease-associated pathways, and nucleotide metabolism (Figure 5F). Following EB administration, significant pathway enrichment was observed for nucleotide metabolism, biosynthesis of amino acids, pyrimidine metabolism, arginine and proline metabolism, and purine metabolism (Figure 5G). After 4 weeks of combined TFA + EB treatment, enriched pathways included α-linolenic acid metabolism, Parkinson’s disease pathways, lysine biosynthesis, inflammatory mediator regulation of TRP channels, and nucleotide metabolism (Figure 5H). Notably, cortisol levels were significantly elevated in the DN group and were associated with steroid hormone biosynthesis pathway enrichment. TFA treatment effectively reduced cortisol levels in DN mice. Additionally, xanthine and inosine showed dramatic upregulation, correlating with purine metabolism and nucleotide metabolism pathway enrichment, whereas thymidine was specifically associated with nucleotide metabolism pathway activation (Figures 5E–H).

### 3.6 Gut microbiota–serum metabolome correlation network

**Figure 6:**
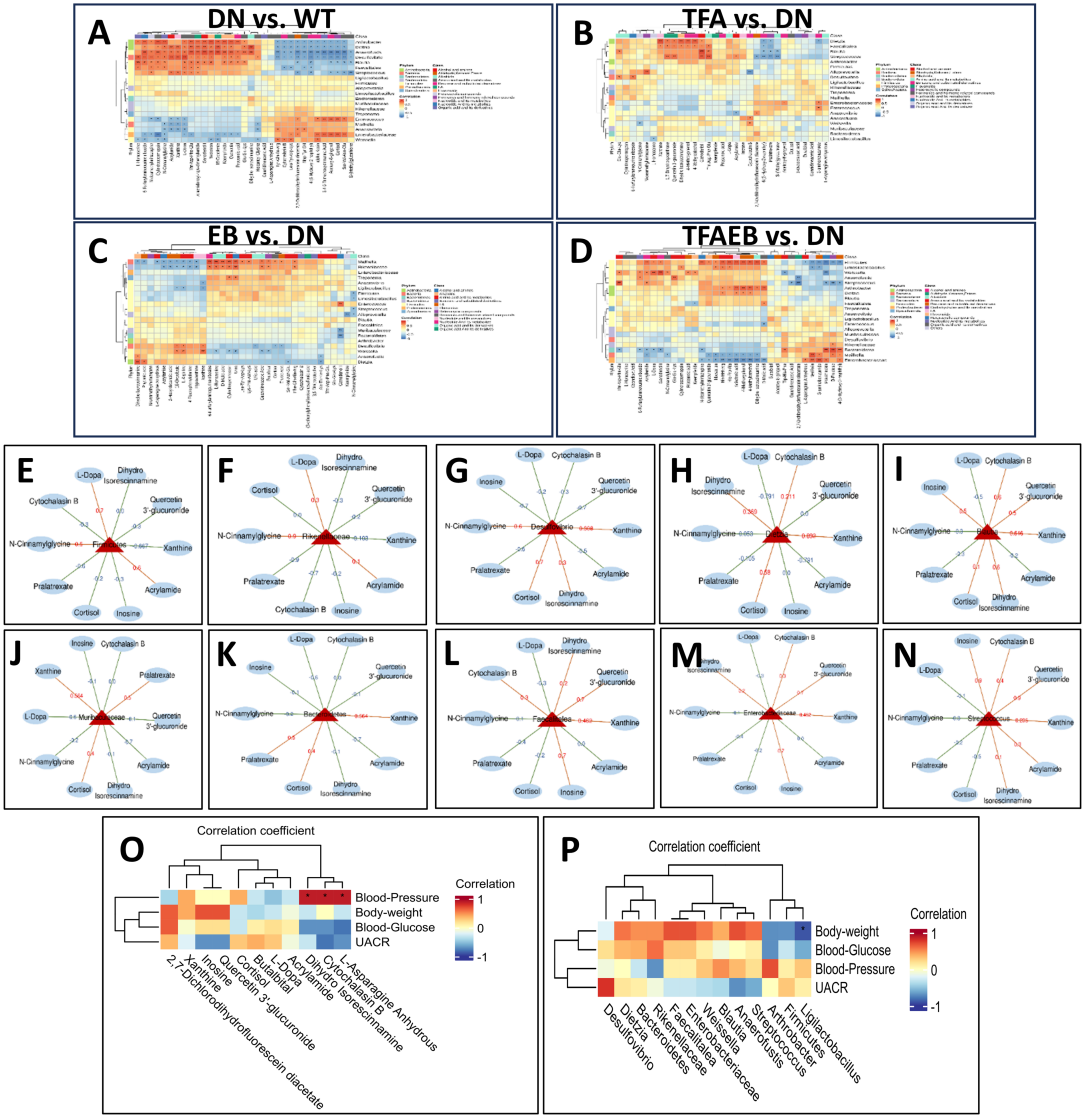
Correlation between the changed gut microbiota, altered serum metabolites, and clinical parameters. Cluster heatmap of Spearman’s correlation analysis of metabolites and microorganisms in the DN, TFA, EB, and TFAEB groups **(A–D)**. The correlation analysis of the main metabolites with the gut microbiota shows that the red line represents a positive correlation, while the green line represents a negative correlation **(E–N)**. Spearman’s correlation analysis was performed to evaluate the associations between physiological and biochemical indices (blood glucose, body weight, blood pressure, UACR, both metabolites, and gut microbiota) **(O,P)**. The results highlight significant correlations (*p* < 0.05) between specific metabolic pathways (e.g., amino acid metabolism and lipid metabolism) and key bacterial taxa (e.g., Lactobacillus and Bacteroides) with disease-relevant clinical parameters, providing insights into potential microbial and metabolic contributors to metabolic dysfunction. DN, diabetic nephropathy; WT, non-diabetic control; TFA, total flavones of *Abelmoschus manihot* (L.); EB, irbesartan; TFAEB, TFA combined with EB. UACR, urinary albumin-to-creatinine ratio.

The clustering heatmap displays Spearman’s correlation analysis between gut microbiota and serum metabolites in db/db mice following 4-week treatments with TFA, EB, and their combination (Figures 6A–D). Firmicutes and Rikenellaceae showed positive correlations (red) between L-dopa, acrylamide, and N-cinnamylglycine while exhibiting negative correlations (blue) between quercetin 3′-glucuronide and pralatrexate (Figures 6E,F). Bacteroidetes, Desulfovibrio, Blautia, and Muribaculaceae exhibited positive correlations between cortisol and xanthine but negative correlations between L-dopa, acrylamide, and quercetin 3′-glucuronide (Figures 6G–K). Notably, Faecalitalea, Enterobacteriaceae, and Streptococcus were positively correlated with quercetin 3′-glucuronide and inosine while showing negative correlations with both L-dopa and cortisol (Figures 6L–N). As shown in Figures 6O,P, Spearman’s correlation analysis was performed to assess the relationships between physiological and biochemical indicators (blood glucose, body weight, blood pressure, and UACR) and metabolites/gut microbiota following treatment with TFA, EB, or their combination. The heatmap revealed that body weight, blood glucose, and UACR were not significantly correlated with any metabolites. In contrast, blood pressure exhibited positive correlations with three specific metabolites: dihydro isorescinnamine and L-asparagine anhydrous. Additionally, body weight showed a positive correlation with the gut bacterial genus Ligilactobacillus, while no significant associations were observed between blood pressure, blood glucose, or UACR and metabolites.

### 3.7 Renal transcriptomic responses to TFA, EB, or combination

**Figure 7:**
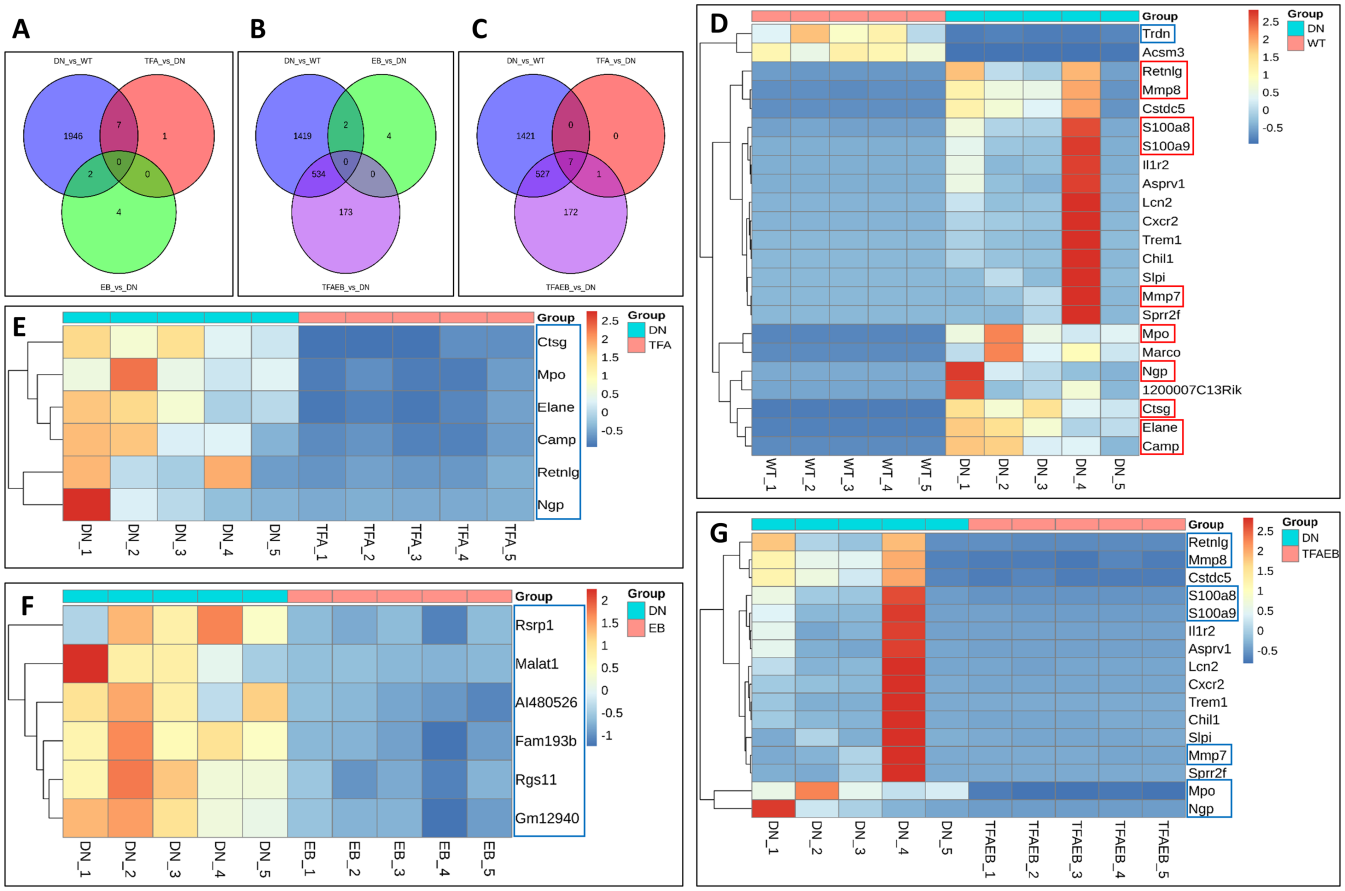
Genes differentially expressed in kidneys. Venn diagrams revealed common and unique genes in kidney tissues after TFA, EB, and TFAEB treatments **(A–C)**. Cluster heatmaps display the change of kidney gene in all treatment groups **(D–G)**. DN, diabetic nephropathy; WT, non-diabetic control; TFA, total flavones of *Abelmoschus manihot* (L.); EB, irbesartan; TFAEB, TFA combined with EB. The red box indicates upregulation, while the blue box means downregulation.

The Venn diagram illustrates common and unique genes in kidney tissues across the experimental groups, with 1,966, 8, 6, and 707 genes detected in the DN, TFA, EB, and TFAEB groups, respectively (Figures 7A–C). TFA treatment resulted in one significantly increased and seven decreased renal genes. The EB group showed 6 downregulated kidney genes, while combined TFA + EB treatment induced 197 upregulated and 610 downregulated genes. Cluster heatmaps visualized DEG patterns across all treatment groups (Figures 7D–G). In the DN groups, upregulated genes included Retnlg, Mmp8, S100a8, S100a9, Mmp7, Ngp, Mpo, Camp, Ctsg, and Elane, with Trdn being downregulated (Figure 7B). TFA treatment significantly increased Trdn expression while downregulating Retnlg, Ngp, Mpo, Camp, Ctsg, and Elane (Figure 7C). The EB group exhibited downregulation of Fam193b, Malat1, Rgs11, Gm12940, Rsrp1, and AI480626 (Figure 7D). Notably, combined treatment downregulated Retnlg, Mmp8, S100a8, S100a9, Mmp7, Ngp, and Mpo (Figure 7E).

### 3.8 KEGG pathway enrichment of differentially expressed renal genes‌

**Figure 8:**
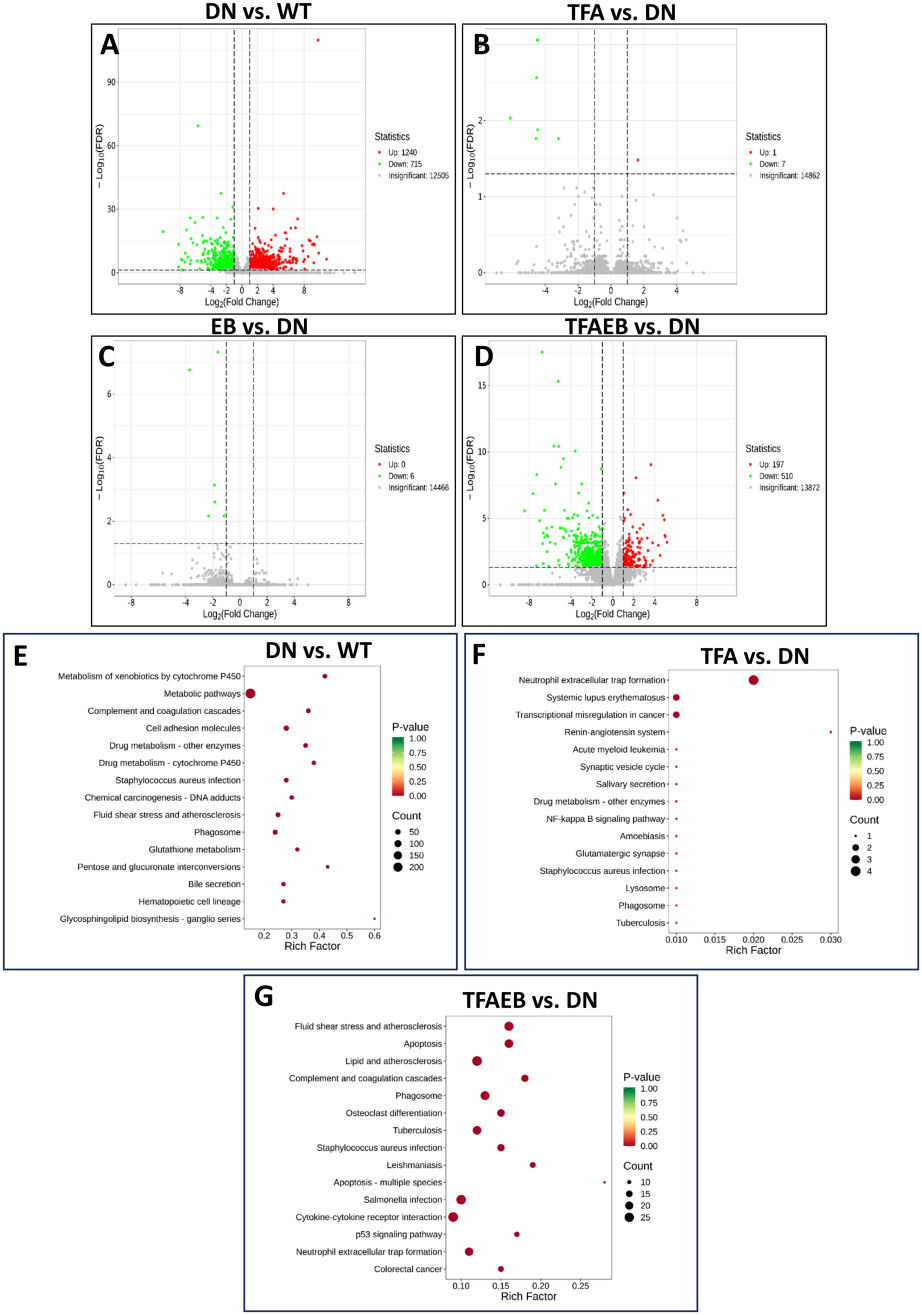
Volcano plot displays a KEGG pathway enrichment analysis of differential genes in each group. The volcano plot displays the common and distinct genes in the kidney among the groups **(A–D)**. Further KEGG pathway analysis of the renal gene of each group in DN compared to WT **(E)** and after the treatment of TFA and TFAEB **(F,G)** is represented. DN, diabetic nephropathy; WT, non-diabetic control; TFA, total flavones of *Abelmoschus manihot* (L.); TFAEB, TFA combined with EB.

The volcano plot displays the common and distinct genes in the kidney among the groups (Figures 8A–D). The pathways that were significantly enriched in the DN group included metabolic pathways, metabolism of xenobiotics by cytochrome p460, cell adhesion molecules, glutathione, and pentose and glucuronate interconversions (Figure 8E). The neutrophil extracellular trap formation pathway was significantly enriched in the TFA group (Figure 8F). In the TFAEB group, the fluid shear stress and atherosclerosis, apoptosis, lipid and atherosclerosis, cell adhesion molecules, and phagosome were enriched (Figure 8G). In the EB group, no pathway was found. The differential genes in the kidney, including Elane, MPO, and Ctsg, were downregulated and were enriched in the neutrophil extracellular trap formation pathway after the TFA treatment for 4 weeks. Furthermore, Itgb3 and Il1r2 were downregulated and enriched in the lipid and atherosclerosis pathways after TFA and EB combination administration.

### 3.9 Integrated analysis of renal gene expression and serum metabolomics

**Figure 9:**
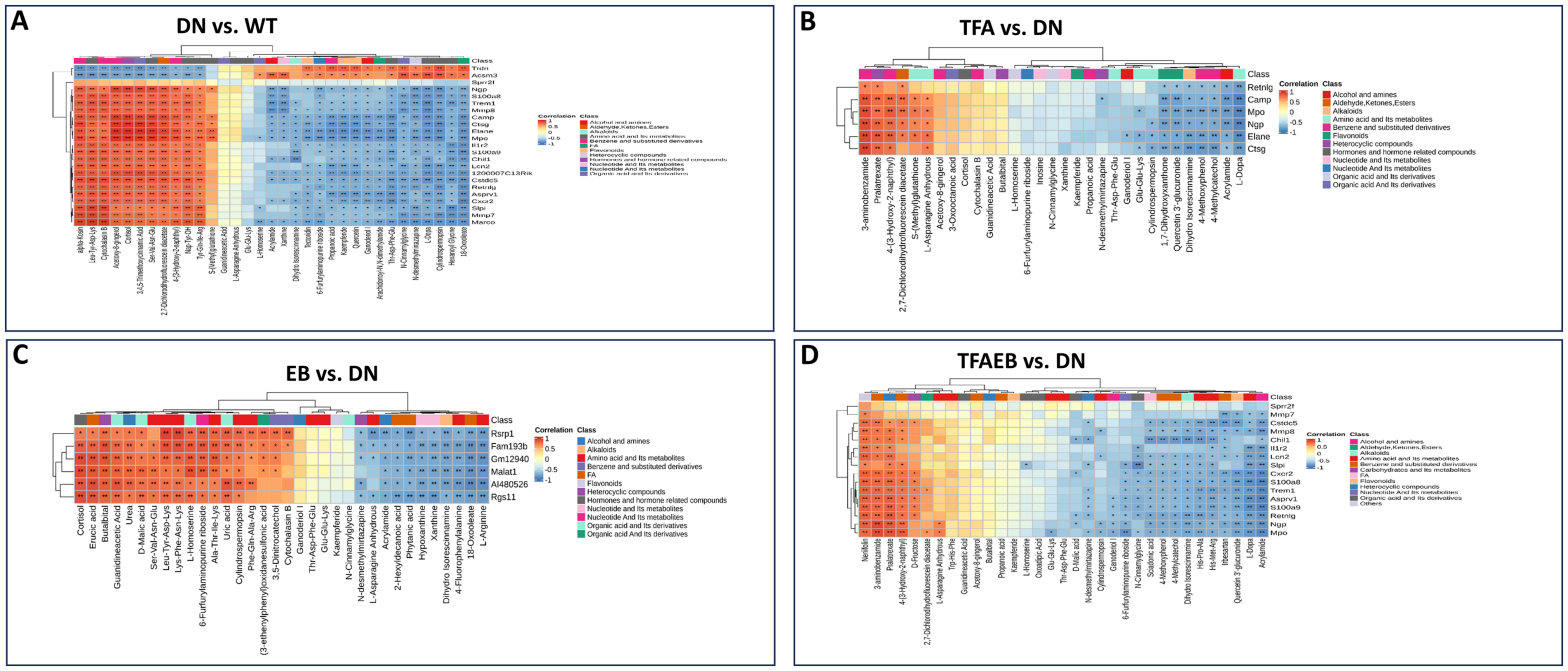
Correlation between the changed gene and altered serum metabolites. The relationship between the changed renal gene and varied serum metabolites in DN compared to WT **(A)**, and after the treatment of TFA, EB, or TFAEB, was shown **(B–D)**. WT, non-diabetic control; TFA, total flavones of *Abelmoschus manihot* (L.); EB, irbesartan; TFAEB, TFA combined with EB.

**Figure 10:**
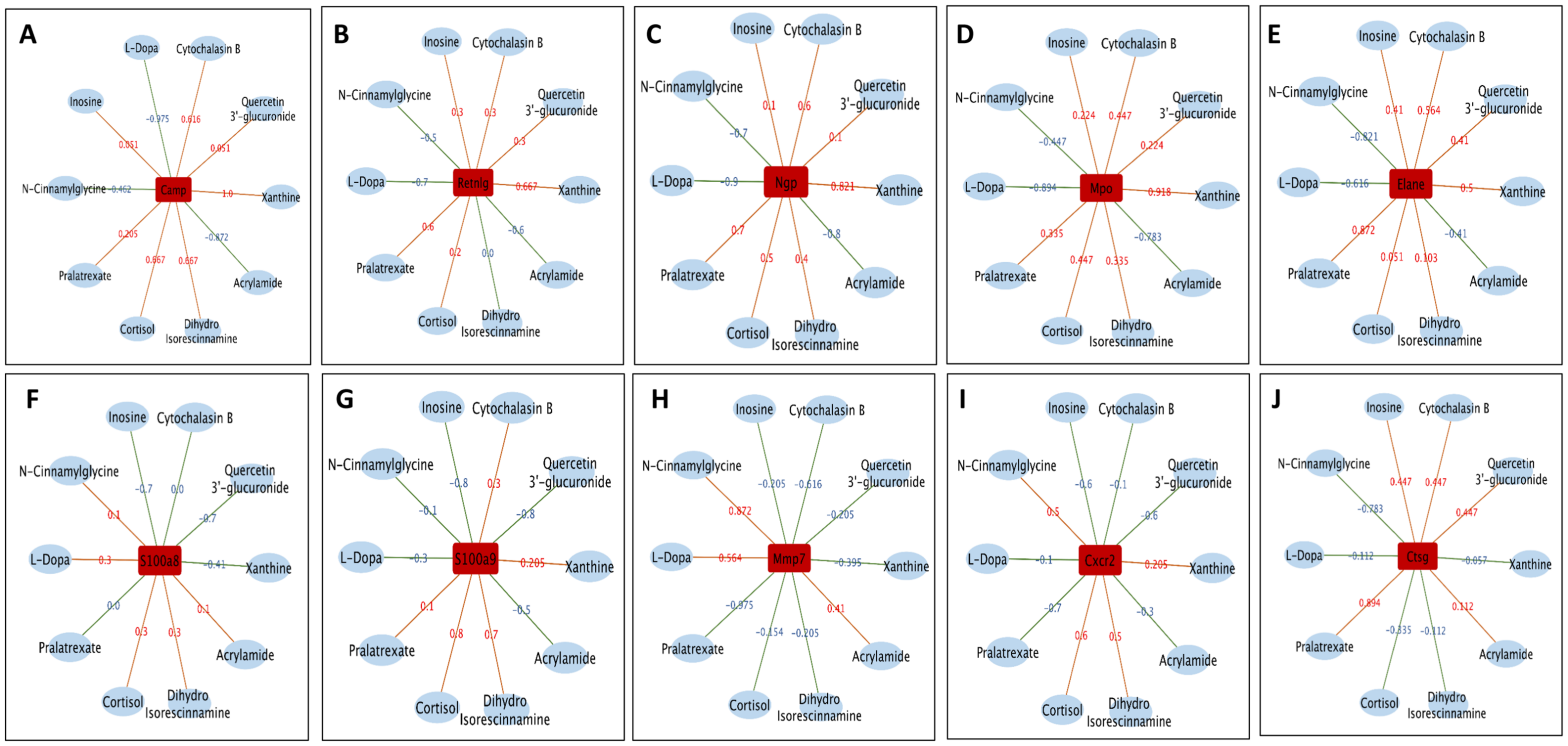
Correlation between the changed genes and altered serum metabolites. The major renal genes are shown, and each is correlated with several serum metabolites **(A–J)**. The red line means positively correlated, while the blue line is negative. DN, diabetic nephropathy; WT, non-diabetic control; TFA, total flavones of *Abelmoschus manihot* (L.); EB, irbesartan; TFAEB, TFA combined with EB.

Spearman’s correlation analysis was performed between renal gene expression and serum metabolite levels across the DN vs. WT, TFA vs. DN, EB vs. DN, and TFAEB vs. DN comparison groups. Clustering heatmaps depicting these correlations are presented in Figures 9A–D. Specific correlation patterns between key genes and metabolites were identified: Camp, Ngp, Elane, and Mop showed positive correlations between quercetin 3′-glucuronide, xanthine, dihydro isorescinnamine, cortisol, pralatrexate, and inosine, but they showed negative correlations between L-dopa, acrylamide, and N-cinnamylglycine (Figures 10A–E). S100a8 was positively correlated with L-dopa, cortisol, dihydro isorescinnamine, acrylamide, and N-cinnamylglycine and was negatively correlated with quercetin 3′-glucuronide, xanthine, pralatrexate, and inosine (Figure 10F). S100a9 exhibited positive correlations between cortisol, xanthine, dihydro isorescinnamine, and pralatrexate and exhibited negative correlations between quercetin 3′-glucuronide, acrylamide, L-dopa, and N-cinnamylglycine (Figure 10G). Mmp7 was positively correlated with L-dopa, acrylamide, and N-cinnamylglycine and was negatively correlated with quercetin 3′-glucuronide, xanthine, dihydro isorescinnamine, cortisol, pralatrexate, and inosine (Figure 10H). Cxcr2 showed positive correlations between cortisol, dihydro isorescinnamine, xanthine, and N-cinnamylglycine and negative correlations between quercetin 3′-glucuronide, L-dopa, acrylamide, pralatrexate, and inosine (Figure 10I). Finally, Ctsg exhibited positive correlations between quercetin 3′-glucuronide, acrylamide, pralatrexate, and inosine and negative correlations between cortisol, dihydro isorescinnamine, N-cinnamylglycine, and xanthine (Figure 10J).

## 4 Discussion

Our experimental study has systematically investigated the therapeutic mechanisms of TFA, EB, and their combination in treating DN through the gut–kidney axis. The administration of TFA demonstrated significant therapeutic effects, notably reducing the UACR in DN models. Microbial analysis revealed that pathogenic genera including Weissella, Alloprevotella, Mailhella, Treponema, Enterobacteriaceae, Rikenellaceae, Enterococcus, and Desulfovibrio, which were abnormally elevated in DN, showed marked reduction following TFA treatment. Concurrently, we observed normalization of serum biomarkers, with cortisol and cytorelaxin B levels decreasing significantly post-treatment. Renal gene expression profiling demonstrated downregulation of multiple inflammatory markers, including Retnlg, Ngp, Mpo, Camp, Ctsg, Elane, S100a8, S100a9, Trem1, and Mmp7, corroborating our previous findings on human kidney cells (HKC) and confirming TFA as the bioactive component of *A. manihot* (L.) in DN treatment (12, 14, 15), primarily through gut–kidney axis modulation.

The intricate interplay between gut microbiota and serum metabolites (15, 17) was particularly evident in our DN models. We documented significant enrichment of Bacteroidetes and Firmicutes phyla in DN mice, alongside depletion of Muribaculaceae—findings consistent with clinical observations in DN patients (16). Spearman’s correlation analysis established significant positive associations between specific bacterial taxa and cortisol levels, while renal transcriptomics implicated steroid hormone biosynthesis and cortisol synthesis/secretion pathways in DN pathogenesis.

The glucocorticoid pathway emerged as a critical mediator in DN progression, with cortisol demonstrating particularly strong clinical correlations. Elevated serum cortisol, a well-characterized glucocorticoid (18), was observed in both our DN models and human studies (19), showing a positive correlation with proteinuria. Mechanistically, glucocorticoid overactivation exacerbates insulin resistance through multiple pathways (20, 21), promoting renal fibrosis and glomerulosclerosis (22). Our findings revealed that TFA treatment significantly reduces cortisol levels in db/db mice, suggesting its therapeutic potential in modulating steroid hormone biosynthesis and cortisol secretion pathways to halt DN progression.

Our findings revealed that TFA administration induces significant alterations in serum metabolites that subsequently modulate renal gene expression. Notably, we identified a positive correlation between renal cxcr2 gene expression and serum cortisol levels. This observation aligns with existing evidence that cxcr2 deficiency ameliorates renal inflammation in DN through suppression of the NF-κB signaling pathway (23, 24). Conversely, mmp7 and ctsg expression showed negative correlations with cortisol. These findings are particularly significant, as mmp7 has been established as both a diagnostic biomarker for renal fibrosis and a predictor of future renal function decline (25, 26). Importantly, TFA treatment significantly downregulated renal expression of both mmp7 and cxcr2 in db/db mice, suggesting a potential molecular mechanism involving: (1) modulation of colonic Bacteroidetes and Muribaculaceae populations, (2) regulation of cortisol expression through steroid hormone biosynthesis pathways, and (3) consequent effects on renal cxcr2 and mmp7 expression.

Further analysis revealed a positive correlation between renal MPO gene expression and serum xanthine levels. Both MPO and xanthine oxidase are recognized as key mediators of reactive oxygen species (ROS) production (27), with excessive ROS contributing to oxidative stress, inflammatory infiltration, and subsequent glomerular/tubular damage in DN (21, 22). The xanthine metabolic pathway generates ROS that may activate mmp7 expression, while urinary mmp7 has been identified as a sensitive biomarker for renal injury (28). These observations suggested an additional therapeutic mechanism whereby TFA may attenuate DN progression by: (1) reducing gut Desulfovibrio and Blautia abundance, (2) modulating serum xanthine levels, and (3) downregulating renal MPO and mmp7 expression.

While the angiotensin receptor blocker EB exhibited renoprotective effects in our study, evidenced by reduced UACR, we observed no significant impact on blood pressure or specific renal gene expression pathways. This finding is consistent with clinical studies showing EB’s blood pressure-independent renoprotection in T2D patients with microalbuminuria (29, 30). A notable limitation of our study stems from the chemical complexity of TFA, which contains seven distinct flavones (10, 31), making comprehensive pharmacological investigation of individual components and their combinations challenging. While our findings establish gut–kidney axis regulation as the fundamental mechanism of TFA’s therapeutic effects in DN, further research is warranted to elucidate the specific pathways modulated by each TFA component.

Traditional Chinese medicine (TCM) improves chronic kidney disease (CKD) by regulating intestinal flora and metabolic disorders, as evidenced by studies on anthraquinones from *Rheum officinale* ameliorating renal fibrosis in acute and CKD (32)‌ and clinical trials showing Shenkang suppository’s efficacy in delaying CKD progression by modulating gut microbiota dysbiosis. CKD is closely linked to gut dysbiosis, which exacerbates intestinal inflammation, oxidative stress, and systemic translocation of uremic toxins (e.g., indoxyl sulfate and phenyl sulfate) (33, 34), thereby aggravating renal fibrosis. Interventions such as *Bifidobacterium bifidum* tablets and Jin Gui Ren Qi Pill have shown promise in restoring gut flora and metabolic balance in DN patients. Mechanistically, gut microbiota-derived metabolites (e.g., indole-3-propionic acid) mitigate DN by enhancing mitochondrial protection and reducing SIRT1 degradation (35, 36). Additionally, the kidney–gut–brain axis highlights the interplay between gut dysbiosis, inflammation, and oxidative stress in CKD progression (37). These findings underscore the potential of TCM and microbiota-targeted therapies in CKD management.

## 5 Conclusion

This study provides compelling evidence that TFA from *A. manihot* (L.) effectively attenuates DN progression through multi-target mechanisms involving intestinal microbiota modulation, circulating metabolite regulation, and renal gene expression control. The finding further advances our understanding of TFA’s therapeutic potential while highlighting important directions for future medical application of *A. manihot* (L.) in the treatment of renal diseases.

## Data Availability

The datasets presented in this study can be found in online repositories. The names of the repository/repositories and accession number(s) can be found below: https://www.ncbi.nlm.nih.gov/, PRJNA1120169.
